# Emerging plant disease epidemics: Biological research is key but not enough

**DOI:** 10.1371/journal.pbio.2007020

**Published:** 2018-08-22

**Authors:** Rodrigo P. P. Almeida

**Affiliations:** Department of Environmental Science, Policy and Management, University of California, Berkeley, Berkeley, California, United States of America

## Abstract

The effective management of plant diseases is of fundamental importance for food production, forestry, and other plant-derived products, as well as for the sustainability of natural environments. When considering the impact of a plant pathogen, the financial costs incurred by an outbreak usually receive the most focus, but there are other much less understood consequences for the affected society, culture, and environment due to disease. This poorly studied layer of complexity is particularly relevant for emerging outbreaks, of which often only limited knowledge is available to devise management strategies, but decisions and actions must be made quickly. The recent outbreak of a bacterial plant pathogen in Europe illustrates how understanding not only the biology of an emerging pathogen but also the cultural context is critical for effectively communicating and engaging with stakeholders and policy makers in order to implement successful disease control strategies.

Plant pathogens and diseases are integral components of ecosystems; however, plant pathology research is significantly biased toward agriculture and forestry. Most research in plant pathology focuses on two types of diseases: those that are established and actively managed and those that are emerging and less well understood. Plant disease emergence is driven by various factors, particularly pathogen introductions into new regions. Pathogen biology and ecology may be difficult to predict in new environments. Previously available knowledge is key to devise eradication, quarantine, and management strategies, but it must be adapted to novel scenarios in short time frames to be effective. While the academic community often rises to the technical challenges of responding to an emerging outbreak, what actually happens during early stages of new disease epidemics is also strongly influenced by political, economical, and societal pressures. Important decisions such as the pursuit of eradication efforts and how to implement those efforts must be made quickly, decidedly, and may not be entirely supported by the scientific literature. Here, I reflect on the role of science, scientists, and scientific uncertainty in this process based on my experience with an emerging plant disease epidemic.

In October 2013, the bacterium *Xylella fastidiosa* was first reported in Europe when it was identified in infected olive trees in Apulia, Italy [1]. The European Commission immediately requested that an eradication program be implemented, a decision based on perceived regional threats imposed by a quarantine organism. Because this pathogen was not previously reported in Europe, no established local expertise existed. At the time, most *X*. *fastidiosa* research occurred in the United States and Brazil, where the pathogen causes several diseases of economic importance. As such, all knowledge available to decision-makers was coming from outside the affected area, generated in other countries, and being used by the European Commission to impose strong eradication measures in a region of a member state. While the directives from the European Commission were precautionary, science-based, and reasonable, considering their stated goals and the situation on the ground, scientific uncertainty was misused and resulted in a significant backlash in the affected Italian region. This rebuttal ultimately led to a lack of substantive efforts to eradicate or contain the pathogen, resulting in plant loss and devastation at the landscape level, which continues today (Fig 1) [2]. In retrospect, the backlash should have been no surprise, as Apulia produces approximately 30% of Italy’s olive oil in an estimated area of 370,000 hectares, representing an important component of the local economy. Furthermore, centennial olive trees dominate the landscape, attract tourists, are culturally and socially important, and are legally protected.

**Fig 1:**
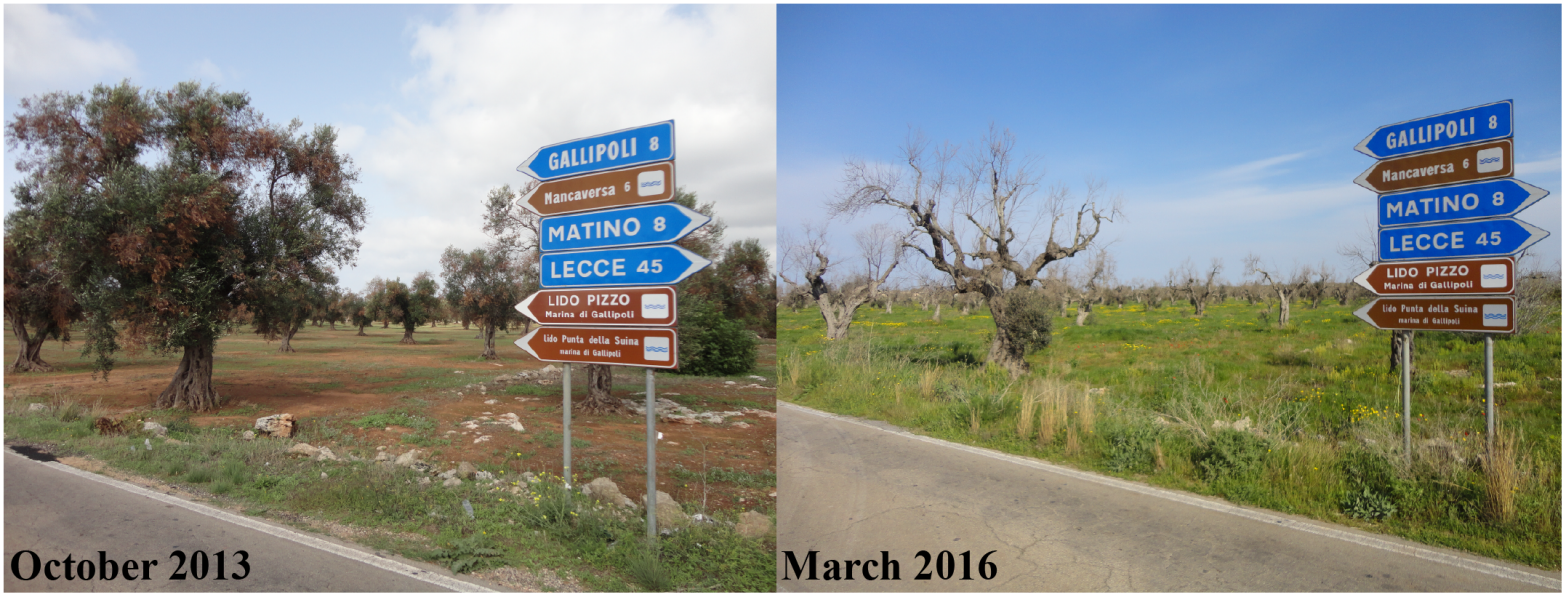
Before and after photographs of an olive grove in the Apulia region of Italy devastated by *Xylella fastidiosa* infection. Photographs kindly provided by Donato Boscia.

*X*. *fastidiosa* is a vector-borne plant pathogen that colonizes a growing list of host plants that already includes over 350 species [3]. Although any strain of the bacterium is capable of colonizing a number of plant species, particular pathogen strain–host plant species combinations are required for the development of symptomatic infections and are of economic importance. Once a plant is infected, there is no cure other than antibiotic applications, and this treatment is not viable at the landscape level. Strategies to cure or limit the impact of *X*. *fastidiosa* infections continue to be investigated, including the disruption of cell–cell signaling and use of bacteriophages. While cures are sought, plant pathologists, entomologists, and ecologists attempt to devise management strategies. Many vector-borne pathogens are highly specific for their vectors, but *X*. *fastidiosa* is unusual in that large groups of xylem sap-sucking insects in two families can act as agents for dispersal, the sharpshooter leafhoppers and spittlebugs. *X*. *fastidiosa* colonizes the mouthparts of these insects in a persistent manner without any degree of specificity, meaning that, so far, any species in these two large groups should be considered a vector. In practice, that means that vectors are already available in most regions of the world, only requiring the introduction of a bacterial strain that is pathogenic to a local crop for novel epidemics to develop. Research integrating knowledge from various disciplines has led to the development of disease-specific control strategies for the better studied diseases, such as approaches for vector suppression and the use of *X*. *fastidiosa* tolerant or resistant plant varieties.

One interesting facet of the epidemic in Italy was how science and scientific facts were effectively distorted and manipulated to feed pseudoscientific theories disseminated in mainstream and social media platforms to discredit available information used to formulate control strategies and to discredit scientists, ultimately leading to the placement of researchers themselves under investigation by a local prosecutor [4,5]. Similarly to challenges still faced by other academic disciplines, the scientifically appropriate acknowledgment of limitations was twisted to mean insufficient availability of knowledge, allowing for the seeds of doubt to be sown and alternative narratives to be developed. For example, lack of a near perfect correlation between disease symptoms and detection of the pathogen was used to suggest that *X*. *fastidiosa* may not be associated with the olive disease. The demonstration of Koch’s postulates (an established strategy sufficient to show that a culturable pathogen is the causative agent of a disease) could not be quickly completed to provide the proof needed. Even once Koch’s postulates were fulfilled and published in a peer-reviewed journal [6], questions about sample size and other details were raised to challenge the reliability of the results.

The ability of those discrediting science to develop alternative narratives that then became ingrained in the local population was remarkable and remains in stark contrast to the inability of the scientists to adequately communicate the basic facts bearing on this epidemic [1]. It also points to a general lack of trust in science. There is also a dearth of research studying social and cultural components of plant disease epidemics. Furthermore, the biologists studying the plant pathogens often do not know how people impacted will respond to epidemics or the wider implications beyond loss of yield or environmental disruption. Scientists need a working knowledge of how to effectively engage with affected stakeholders. These are glaring research gaps that must be addressed by the plant pathology community. If we have no research or training on how to best communicate science-based information to stakeholders and decision-makers, ad-hoc communication and outreach efforts by plant pathologists already overburdened by the work of researching an emerging pathogen are certainly bound to fail.

Conventionally, scientists have not been trained on how to communicate their science effectively to the public, although this is changing. For example, existing and new requirements by funding agencies have started to address this problem. When scientists do engage, we often have difficulty in translating our research to an accessible, coherent, and appealing format. I confess to having limited knowledge as to how that predicament can be improved, but various academic programs as well as societies have started to recognize this problem. The American Phytopathological Society, for example, has workshops on communicating science. While an excellent start, it will take time and effort for the community to master the skill of communicating science-based recommendations to the public. Lastly, being involved in public debates can be frustrating as well as potentially unsettling. In the case of the Italian olive disease epidemic, for example, I was accused of deliberately introducing the pathogen in one early narrative. This accusation was made despite knowledge of the source of the pathogen introduction and was most likely made to discredit scientists and science, but principally, the eradication protocols recommended to limit pathogen spread. While this anecdote may sound amusing, it occupied my mind more than it should have. In actuality, phylogenetic data demonstrated that the olive disease strain was a single introduction originated from Central America (e.g., [7]). In fact, early studies on this question led to the interception of several shipments into European ports with *X*. *fastidiosa*-infected plants and ultimately to significant regulatory changes affecting international trade [1]. Thus, research-based evidence did impact world trade of ornamental plants and will hopefully limit the likelihood of future pathogen introductions.

In the context of plant disease epidemics—but certainly also other environmental challenges and beyond—despite the drawbacks, I argue scientists have to step up to the plate. We must continue to pursue research on pressing issues, regardless of political, economic, and other pressures. Studying disease spread patterns is required to devise pathogen quarantine and containment strategies. Fundamental science is needed, as no curative approaches can be developed without understanding how pathogens interact with host plants and cause disease. Additionally, the identification of insect vectors is mandatory to suppress plant-to-plant dissemination of vector-borne pathogens. To be clear, the biological research matters, as it is key to our understanding of the natural world and, consequently, to ultimately develop sound plant disease management approaches. However, in the case of emerging epidemics, research on plant pathogens and diseases are not everything. Consideration of the human populations affected by plant disease is also essential, as is effectively communicating scientific data and uncertainties. Listening and learning to understand the range of concerns and priorities of diverse stakeholders must be part of this process. These stakeholders include governmental agencies that are often staffed by scientists. But we must not forget other stakeholders, especially the public and the politicians. Engaging with the media can be nerve wracking, and it will inevitably lead to misquotations and decontextualization, deliberate or not. But we must be willing to do it, not as infallible experts or activists but as individuals who have particular knowledge that should be considered in public debates. We should support and not denigrate or disparage the efforts of colleagues that engage with the public, recognizing that they might over simplify facts or complex concepts and use bad analogies, and we should not accuse them of self-aggrandizement.

If anything, the olive disease outbreak in Europe taught me that research matters; fundamental and applied knowledge on plant diseases is required for any meaningful action to manage emerging epidemics. But the social context of epidemics must also be considered. I learned that I was, and remain, ill prepared and untrained to communicate science to a broad range of stakeholders. Exchanges with a reporter, written or verbal, are unquestionably more difficult and time consuming than those with a scientist. But we must not shy away from such interactions; communicating science is difficult, but it has become an important component of the job of being a scientist. Remaining in the familiar settings of academia is no longer enough if scientists are to have an impact and help shape the future.
